# *Eriocaulon madayiparense* (Eriocaulaceae) – A new species from the foot hills of the Western Ghats of India

**DOI:** 10.3897/phytokeys.10.2297

**Published:** 2012-03-16

**Authors:** M. M. Swapna, K. P. Rajesh, C. N. Manju, R. Prakashkumar

**Affiliations:** 1 Malabar Botanical Garden, G.A. College P.O., Kozhikode-673 014, Kerala, India; 2 The Zamorin’s Guruvayurappan College, G.A. College P.O., Kozhikode-673 014, Kerala, India

**Keywords:** *Eriocaulon madayiparense*, Eriocaulaceae, India, Western Ghats, Madayipara, Laterite hill

## Abstract

*Eriocaulon madayiparense* Swapna, Rajesh, Manju & Prakashkumar, **sp. nov.** is described and illustrated from the Madayipara, a lateritic hillock in the midland of Kannur District of Kerala. The species is allied to *Eriocaulon eurypeplon* Koernicke, in its two free male and female sepals, female sepals being keeled and acute and not exceeding the floral bracts, acuminate leaf apex and setiform seed appendages appearing in vertical rows, but differs mainly in having yellow seeds with solitary appendage arising from transverse radial walls, curved and connate with the adjacent ones of the same vertical row forming longitudinal parallel ribs on the surface of the seeds.

## Introduction

The genus *Eriocaulon* is well represented in India with many endemic species. Ansari and Balakrishnan (2009) provided a detailed account of 80 species in India. However, novelties are being added in the genus in recent years such as *Eriocaulon epedunculatum* Potdar et al. (Yadav et al. (2008), *Eriocaulon baramaticum* Shimpale et al. (2009), *Eriocaulon belgaumensis* Shimpale & Yadav (2010), *Eriocaulon wayanadense* Vivek et al. (2010), *Eriocaulon malabaricum* Pradeep & Nampy and *Eriocaulon pykarense* Nampy & Manudev (Nampy et al. 2011). The present one is another addition, from the foot hills of the Western Ghats of India. It belongs to the section VI proposed by Ansari and Balakrishnan (2009). It is allied to *Eriocaulon eurypeplon* Koernicke, and shows resemblances such as two free male and female sepals, female sepals being keeled and acute and not exceeding the floral bracts, acuminate leaf apex and setiform appendages appearing in vertical rows. However it strongly differs in having black coloured, glabrous, acute-acuminate involucral bracts, female sepals being irregularly toothed towards apexat back, unequal petals and yellow seeds with solitary appendage arising from transverse radial walls, curved and connate with the adjacent ones of the same vertical row forming longitudinal parallel ribs on the surface of seeds (Table 1). Hence it is described here as a new species.

**Table 1.** Comparison between *Eriocaulon eurypeplon* and *Eriocaulon madayiparense*

Characters	*Eriocaulon eurypeplon*	*Eriocaulon madayiparense*
Involucral bract	Obtuse-subacute, minutely hoary dorsally, straw coloured	acute-acuminate, glabrous, black
Female flower	Pedicels glabrous	Pedicels hairy
Sepals	obovate, acute or obtuse, entire	oblance-ovate or oblanceolate, irregularly toothed towards apex at back
Petals	equal	subequal
Seeds	dark purple, appendages 1-2 from transverse radial walls, free, setiform, dilated at apex	Yellow, appendages solitary from transverse radial walls, curved and connate with the adjacent ones of the same vertical row forming longitudinal parallel ribs on the surface of seeds

### Eriocaulon madayiparense

Swapna, Rajesh, Manju & Prakashkumar sp. nov.

urn:lsid:ipni.org:names:77118190-1

http://species-id.net/wiki/Eriocaulon_madayiparense

#### Diagnosis.

*Eriocaulon madayiparense* is allied to *Eriocaulon eurypeplon* Koernicke, but differs mainly in having black, glabrous, acute-acuminate involucral bracts, pedicels of female flowers hairy at base, female sepals oblance-ovate or oblaceolate, cuneate, conduplicate, keeled, irregularly toothed towards apex, yellow seeds with solitary appendage arising from transverse radial walls, curved and connate with the adjacent ones of the same vertical row forming longitudinal parallel ribs on the surface of seeds.

#### Type.

**INDIA.** Kerala, Kannur District, Madayipara, 40 m alt., 5 September 2011, *K.P.Rajesh & C.N.Manju* 5610 (Holotype: MBGS! Isotypes: MBGS!, MH!, CALI!, K!)

#### Description.

**Figure 1.**
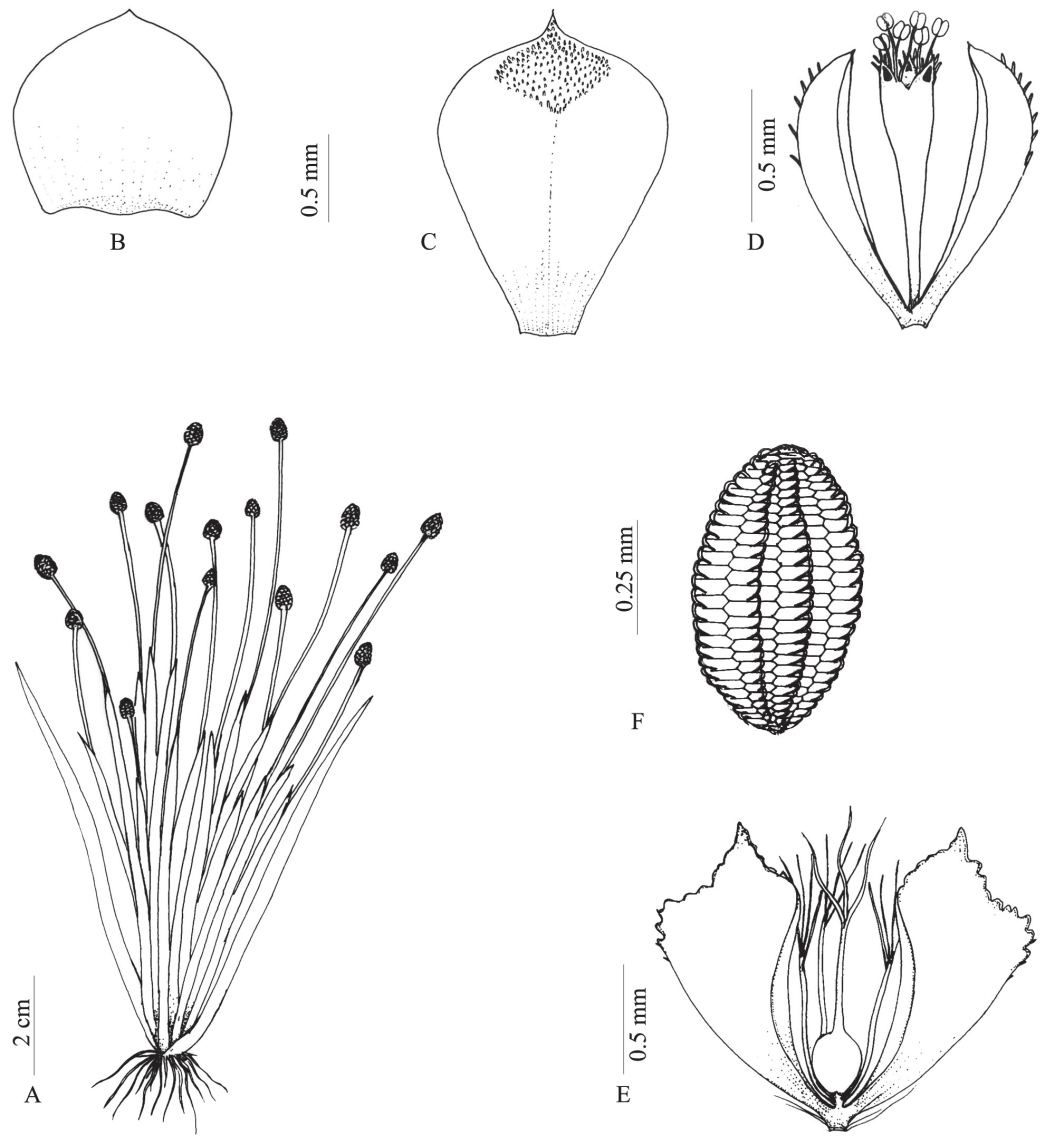
*Eriocaulon madayiparense*, **A** habit **B** Involucral bract **C** Floral bract **D** Male flower **E** Female flower **F** Seed

Acaulescent herbs. Root stock absent. Leaves linear-lanceolate, acute or acuminate, ca. 10–12 × 0.5–0.7 cm, glabrous. Peduncles erect, 2- many, ca. 7–13 cm long, rigid, glabrous. Sheaths ca. 5–7 cm long, glabrous; limb ovate, acuminate, entire. Heads globose-ovate, ca. 8 × 6 mm, grey. Receptacle cylindrical, sparsely pilose. Involucral bracts erect, ovate or obovate, acute-acuminate, ca. 1.1 × 1.2 mm, chartaceous, black. Floral bracts closely imbricated, obovate, base cuneate, apex acuminate, ca. 1.8 × 1.3 mm, coriaceous, minutely hoary dorsally towards apex, black. *Male flowers:* Pedicels minute. Sepals 2, free, oblanceolate, obtuse or acute, keeled, ca. 1.2 mm long, minutely hoary along keels towards apex, black. Stipe of corolla ca. 0.9 mm long. Petals 3, equal, minute, oblong, minutely hoary and minutely toothed at apex with a black gland, anthers 6, black. *Female flowers:* Pedicels minute, hairy at base. Sepals 2, free, similar, oblanceolate-ovate or oblanceolate, cuneate, conduplicate, keeled, irregularly toothed towards apexon keel,ca. 1.5 mm long, minutely hoary along keels towards apex, black. Petals 3, linear, subequal, ca. 0.6 to 0.8 mm long, hyaline, barbate towards apex, with or without a black gland, not stipitate between sepals and petals. Ovary sessile, globose, stigmas 3. Seeds ca. 0.6 × 0.3 mm, yellowish, cells of seed coat transversely elongated, aligned in vertical rows, appendages solitary from the middle of the transverse radial walls, curved and connate with the adjacent ones of the same vertical row forming longitudinal parallel ribs on the surface of the seed (Fig. 1 A–F).

#### Distribution.

 It is distributed in the laterite hills of Northern Kerala in Peninsular India.

#### Ecology.

 The specimens were collected from the Madayipara, a lateritic hillock in Kannur District of Kerala, at latitude 12°2'N and longitude 75°16'E (12°2'N, 75°16'E), and with an altitude of 40–47 msl. It is a notable area being the type locality of some narrow endemics such as *Nymphoides krishnakesara* (Joseph and Sivarajan 1990), *Rotala malabarica* (Pradeep et al. 1990), *Justicia ekakusuma* (Pradeep et al. 1991)and *Lepidagathis keralensis* (Madhusoodanan and Singh 1992). The lateritic outcrops of this area support a grassland-scrub system, unique in its ecology, being active along with the monsoon rains, supporting rich assemblages of many aquatic and semi-aquatic plants and animals, and remaining as barren land as the rain recedes (Palot and Radhakrishnan 2002, 2005, Balakrishnan et al. 2010). More than 500 species of plants were recorded from the area, which includes 59 Peninsular Indian endemics, of which 14 are narrow endemics of Kerala state, confined to the laterite hillocks (Balakrishnan et al. 2010). *Eriocaulon* is also well represented in Madayipara with five species, *viz*., *Eriocaulon cuspidatum*, *Eriocaulon heterolepis*, *Eriocaulon lanceolatum*, *Eriocaulon parviflorum* and *Eriocaulon xeranthemum*. The present species is growing in wet areas of Madayipara, along with species such as *Utricularia reticulata*, *Rotala malabarica*, *Rotala malampuzhensis*, *Rhamphicarpa longiflora*, *Oryza rufipogon*, etc.

#### Flowering and fruiting.

 August–December.

#### Etymology. 

The species is named after the type locality, Madayipara.

#### Conservation status.

 The laterite hillocks in general and the Madayipara in particular are facing high degree of danger of habitat degradation due to ignorance by the public and administrators. The areas are being heavily converted to building sites, mining grounds, dumping sites, etc. The uncontrolled tourist activities are also damaging this fragile ecosystem. The present species, like other narrow endemics mentioned earlier, is also confined to the seasonal pools or wet areas of the lateritic hills. The habitat may be lost irrecoverably, if proper conservation measures are not taken.

#### Specimens examined.

 INDIA, Kerala, Kannur District, Madayipara, 40 m alt., 5 September 2011, *K.P.Rajesh & C.N.Manju* 5610.

## Supplementary Material

XML Treatment for Eriocaulon madayiparense
